# Measuring student motivation on the use of a mobile assisted grammar learning tool

**DOI:** 10.1371/journal.pone.0236862

**Published:** 2020-08-28

**Authors:** Nadia Refat, Hafizoah Kassim, Md Arafatur Rahman, Ramdan bin Razali

**Affiliations:** 1 Center for Modern Languages, Universiti Malaysia Pahang, Gambang, Kuantan, Malaysia; 2 Faculty of Computing, IBM CoE and ERAS, Universiti Malaysia Pahang, Gambang, Kuantan, Malaysia; 3 Faculty of Electrical and Electronics Engineering, Universiti Malaysia Pahang, Gambang, Kuantan, Malaysia; Fondazione Ugo Bordoni, ITALY

## Abstract

Language learning is an emerging research area where researchers have done significant contributions by incorporating technological assistantship (i.e., computer- and mobile-assistant learning). However, it has been revealed from the recent empirical studies that little attention is given on grammar learning with the proper instructional materials design and the motivational framework for designing an efficient mobile-assisted grammar learning tool. This paper hence, reports a preliminary study that investigated learner motivation when a mobile-assisted tool for tense learning was used. This study applied the Attention-Relevance-Confidence-Satisfaction (ARCS) model. It was hypothesized that with the use of the designed mobile- assisted tense learning tool students would be motivated to learn grammar (English tense). In addition, with the increase of motivation, performance outcome in paper- based test would also be improved. With the purpose to investigate the impact of the tool, a sequential mixed-method research design was employed with the use of three research instruments; Instructional Materials Motivation Survey (IMMS), a paper-based test and an interview protocol using a semi-structured interview. Participants were 115 undergraduate students, who were enrolled in a remedial English course. The findings showed that with the effective design of instructional materials, students were motivated to learn grammar, where they were positive at improving their attitude towards learning (male 86%, female 80%). The IMMS findings revealed that students’ motivation increased after using the tool. Moreover, students improved their performance level that was revealed from the outcome of paper-based instrument. Therefore, it is confirmed that the study contributed to designing an effective multimedia based instructions for a mobile-assisted tool that increased learners’ motivational attitude which resulted in an improved learning performance.

## 1 Introduction

Grammar is one of the crucial language aspects where students must be able to demonstrate understanding in building sentences, which requires them to use the English language effectively. A big part of proper sentence construction is based on the knowledge of tense, which helps to construct sentences using the correct time reference. Without time aspects, a sentence cannot have an effective meaning. Thus, teaching tenses is a concern for second language teachers where different approaches have been adopted, including conventional methods. Some have even integrated the conventional and new approaches with the use of modern technological support such as a computer or mobile devices [1, 2]. Utilizing the conventional method of teaching grammar would be challenging for teachers to monitor and support students’ progress compared to using integrated approaches, especially with the assistance of computer technology or mobile devices [3].

Mobile devices that refer to gadgets like smartphones, laptops or tablets, are mostly used these days for language learning because of its flexibility. Due to this and with the access to the internet and potential different activities concerning language learning, Mobile-Assisted Language Learning (MALL) has become a significant topic among researchers [4–7]. Such devices enable users to use different types of language learning applications or tools to improve their language skills such as vocabulary, writing, and grammar.

With the advancement and ubiquitous use of mobile devices, many studies have been conducted to examine the effectiveness of using mobile devices for language learning, including grammar learning. One of the issues continuously debated regarding the use of mobile devices for learning the second language is the instructional design of learning materials. Most of the applications highlight its technical soundness but give lesser focus on how the language content, its technicality and overall utilization of the application for motivating instruction should be based on [8]. In MALL, motivation is an important issue, which should be a major concern for instructional designers to consider. Motivation should guide learners to benefit from the application, and designers need to ensure material development can influence the learning process and learning outcomes [9].

In some studies (especially in social science), demographic analysis based on the differences in gender is regarded as an important issue. In such studies, different statistical results are shown between the males and females to draw out the impacts of intervention due to gender differences. Therefore, to present the impact of using the tool on gender-based on its differences, this study also presented an inferential analysis of IMMS.

In previous studies of language learning, it is observed that motivation has been used in order to improve the environment of language learning or to design the materials without following any theoretical model. Moreover, ARCS model was not implemented for grammar learning. In this regard, this study attempted to provide language learners with a mobile-assisted tense tool, developed within the frameworks of MALL, and ARCS Model, which is a theoretical framework for designing motivational instructions in language learning. In other words, the designed tool was implemented to investigate the impact on the learners on their motivational outcome as well as learning performance. In order to explore the result of the impact, this research work followed a mixed method research design where quantitative and qualitative research method were exploited. It was aimed to know that to what extend students felt motivated towards the tool as well as how much their performance improved after the intervention with the tool. It was also explored in this study, that the opinion of the students on their perceptions on using the tool. Therefore, in a single statement, unlike the other studies, the present research work investigated the impact of the tool that was design based on ARCS model on students motivation and learning performance.

## 2 Related works

In many pieces of research, motivation is an important element for structuring learning materials and preparing for learning instruction. When using technological-based learning tools, the success of any application largely depends on their user-friendliness and usability, which could ultimately create motivation. Otherwise, it will lead to poor learning experience if the motivation is obstructed Therefore, educators should attempt to stimulate and sustain student motivation through effective design of the learning materials and its instructions.

At present, grammar learning has been exercised through web-based technologies where tense is a very important component in grammar. There are a few web-based tense learning tools available such as verbs & tenses and English tenses, but the instructional designs seem to neglect learner’s motivational factors [10]. More in details, English Tense is a web-based tool to teach English tense easily, the latest version is 4.2. publisher: dreamBDit. 2. Best tenses and Verbs Tutor is also an android based grammar learning tool that teaches words and tenses in different languages. The latest version of the application is 1.33 and powered by Team SoftTestLab. Besides, studies, which investigated the multimedia-based instructional design of different subjects, also revealed that most of the design did not consider the motivational approach in the designing process [11–13]. As a result, there is a need to design effective instructional materials that can motivate students, especially when technologies such as mobile applications are used.

One of the referred learning models for motivation is John Keller’s (1987) ARCS model, which highlights major motivational components namely Attention, Relevance, Confidence, Satisfaction, aiming for effective instructional design. The model suggests the strategies to assist motivation in learning and also guides the integration of such strategies into formal curricular and teaching design [14]. Making the learning activities interesting and stimulating for learners is the aim of the ARCS model, which ultimately enhances the capability of cognition for meaningful learning. Other motivational models or theories emphasize motivation in learning in general, such as learning performance, or by relating motivation to psychological aspects of learners, such as attitude [15, 16]. ARCS model is therefore relevant and significant in this study as it highlights motivation aspects during the design and development of instructional materials.

### 2.1 MALL application and language learning

Integrating MALL technology (i.e., cell phones, and hand-held devices) in foreign language learning is becoming a common practice in many higher education institutions [17]. The advancement of MALL is evident in different language learning practices, for example, to learn vocabulary, there are different games developed to increase learning performance. Apart from vocabulary learning, other language skills have also been researched using mobile devices, such as speaking skill in which mobile tool applications have been developed to investigate their effectiveness to enhance the speaking ability of students.

A few studies were conducted to explore the effectiveness of using mobile-based grammar learning applications on a different level of students [4–7]. Grammar learning is one of the important areas where mobile devices have been used and studied, but the number of research done is limited. In these studies, the experimental design was employed where grammar lessons with interactive exercises were provided through mobile devices, and the findings revealed positive outcomes on students’ performance and retention [18].

Studies also looked at the utilization of mobile phones in enhancing grammatical knowledge with language skills [19, 20]. One study investigated the effectiveness of using mobile phones as part of students’ English reading skills and grammar knowledge. Reading materials such as short essays, grammar exercises, and quizzes were regularly sent to the students’ mobile phones. It was found that students have both enhanced their reading skills and grammatical knowledge [19]. Another study investigated using mobile phones to record students’ discussions about grammar items, which recordings were later analyzed by the students on grammatical errors they committed during the conversation. The results showed that students who used mobile phones in their lessons did better than those who did not use mobile devices [20]. It was observed in another study that using a mobile-assisted grammar learning application called Grammar Clinic enhanced users’ self-editing capacity in their writing skills. Grammar clinic is a mobile assisted tool to investigates the writing errors. the tool was first piloted in 2011 over the ESl students. Data were collected through written assignments to examine the effect of Grammar Clinic. A semi-structured interview was also conducted to investigate student perceptions of this mobile application as a tool to improve their English writing. The analysis showed that the students perform better in their assignments, which reflected their self-editing capability when using Grammar Clinic. It was also observed that users had positive opinions on the use of the grammar tool for their learning [21].

Hwang and his colleagues conducted a study on writing skill improvement, which proposed a situation learning system on elementary school students with mobile devices. Students carried out their assigned writing tasks in environments familiar to them, such as on the playground in the school compound or within the classroom facilities. This experimental research requested the experimental group to write on common issues or simulating real-life context using mobile devices. The outcome was positive in terms of enhancing grammar knowledge to build proper sentence construction which makes more meaningful sentences to express real-life experiences. Furthermore, based on interviews outcome from both groups, it is revealed that students who use mobile devices are inspired to write more sentences to describe the situation more thoroughly and clearly [22].

Past discussions have revealed the positive effects of utilizing MALL technologies in language learning. However, these studies did not consider motivation based theoretical framework in the design and development of the instructional materials through MALL technologies. Motivation in language learning is a key factor since it determines to learn success or knowledge improvement [23]. Due to its complexity and details, grammar learning requires learners to give more effort and have a positive attitude to achieve the goal of learning [23], and thus, a proper motivational-based instructional design is essential for effective outcomes of learning [24], and thus, a proper motivational- based instructional design is essential for effective outcomes of learning [25]. Considering the above claims, it can be argued that motivation can play a crucial role in effective grammar learning and that, if the proper motivational theoretical framework is adhered to designing the instructions in mobile-based grammar learning tool, it can increase engagement and effectiveness of learning. Thus, the current study aimed to investigate the effects using a mobile-assisted tense learning tool, which considers the instructional design based on the ARCS model to improve students’ motivation to learn present tense with the mobile application.

### 2.2 The ARCS model and language learning performance outcome

The ARCS model aims at developing instructional materials based on motivational factors. Initializing the motivational instructions, it is also needed to diagnose motivational problems in the existing instructions as well as the environment. The model, therefore, guides educators and curriculum designers to improve the environment or materials. Integrating the components of motivation, such as attention, relevance, confidence, and satisfaction, the ARCS model has been widely applied to instructional design processes for bringing the effectiveness of learning outcome [26]. Many studies are conducted on the implementation of the ARCS model in different areas of education.

In a study, the four sub-scales of Keller’s motivational model were applied in learning material design and classroom environment implementation. It was implemented in a traditional Japanese classroom, which was connected with mobile devices and the internet. The lesson was designed to motivate less confident and anxious learners in communication using English in classrooms. The results verified the effects of the model and instruction design in terms of creating motivation among learners. The use of mobile devices to stimulate consciousness regarding language learning, also helped them to be motivated in the classrooms [27].

Another study used the ARCS model in second language learning, which focused on listening skill enhancement that concentrated on person-based skill enhancement. The model has been designed to improve the listening skills of learners through different activities designed based on the four ARCS criteria [28]. In another research, it was also found that the ARCS model was able to enhance motivation among Japanese students at learning English and exposed them to cultural values. The results of the questionnaires and tests revealed the students’ sustained motivation for writing and competence to engage in inter-cultural communication [29]. Kellers’ ARCS model has also been used to examine Persian language learning where material and environment were taken into consideration to motivate the learners. It was observed that using a motivational instruction design is helpful for second language acquisition, which is evident in integrating motivation into instruction to enhance Persian language skills [30].

It is, therefore, assumed that the ARCS model has been influential, resulting in its emergence into different areas of learning. However, there are limited studies on the implementation of the ARCS model in grammar learning, in addition to its exploitation of mobile technology. Hence, it is highly required to investigate the impact of the model in instructional design for grammar learning since grammar is one of the important components for language learning. With the ARCS model, designing tense instruction for mobile users will be helpful for the learners to enhance their performance and motivate them to learn tense.

## 3 Methodology

A sequential mixed-method research design was employed for this study. This research method is well accepted in different research fields for capturing the best result from quantitative and qualitative analysis [30].

### 3.1 Participants

The participants were students from a technical university, who were enrolled in a remedial English language course entitled Fundamental of the English Language. There were 115 (female, n = 76; male, n = 39, average age = 22 years old) students as participants in the study. The students were from the first year and most of them were from computer science, mechanical engineering, and mathematics departments. The students scored below 5 in the entrance English exam therefore, they were to take the fundamental English course to proceed for the next semester. Simple random sampling was applied to select five groups. In the present research work, one group is considered as a control group while the other four groups as the experimental group. In this study, four groups were taken for the experimental group to justify the sample number enough to represent the population. Moreover, this number of students under the groups were taken due to the easy generalization of the population as well as the best evidence for decision making and minimizing biases. Random sampling also makes the assembling easy and minimizes the variations of sample error.

In this study, students’ consent was obtained prior to conducting the study, and they were informed about data confidentiality, and that they would be excluded if they choose not to participate at any stage of the study.

### 3.2 Research instrument

Three research instruments were used in the study;

1. The Instructional Material Motivation Survey (IMMS)
2. Paper-based test
3. An interview protocol

#### 3.2.1 Instructional Materials Motivation Survey (IMMS)

The IMMS was developed by John Keller [26] based on four components of motivation, i.e. Attention, Relevance, Confidence and Satisfaction of the ARCS Motivational Model. IMMS was used to measure student motivation on the instructional design of a tense learning tool. It consists of 10 items, which utilize Likert-type of 5 scales starting from 1 indicating as not true to 5 to mean very true, measuring motivation on instructional design or materials. The 10 items are based on the aforementioned four criteria such as (2 items for attention, 3 for relevance, 2 for confidence and 3 for satisfaction). The internal consistency has been estimated high, that is based on Cronbach’s alpha (see Table 1).

**Table 1:** IMMS Internal consistency estimates reference.

Scale	Reliability Estimate (Cronbach’s alpha)
Attention	0.78
Relevance	0.81
Confidence	0.80
Satisfaction	0.80

#### 3.2.2 Paper-based test

A paper-based test was developed to measure participants’ level of knowledge and competence of the English present tenses after they have used a mobile-assisted tense tool, an instructional tool developed for the study. It was administered as a pretest and posttest. The test which aimed to identify ranges of users from the very basic ones (A1) to the more independent (B1), consists of questions that follow the Common European Framework of Reference (CEFR). The CEFR was referred to standardize the questions according to appropriate levels of students’ grammatical knowledge. The questions are categorized into three types: (1) multiple-choice questions (to know their remembering capability that covers fundamental understanding of grammar items), (2) fill-in-the-blanks with suitable words (capability to analyze grammatical items), and (3) free-hand paragraph writing (to produce simple connected text on topics of the grammatical items learned based on familiar topics).

To validate the test paper, construct validity was first conducted. The test was checked and reviewed by two experts, who are qualified English language instructors with an experience of more than 10 years of teaching grammar and experienced in designing and validating test papers on grammar. They are also skilled in technology-based instructional materials.

#### 3.2.3 Interview protocol

An interview protocol of a semi-structured interview was conducted to explore learners’ perceptions after using the tool, which could not be retrieved only through the survey of IMMS. Therefore, rich and in-depth explanations of motivational issues in using MALL is effective for investigating the qualitative phase of this study, which is conducted after the quantitative data collection. Interviews were conducted to examine four aspects (1) interest of the tool, (2) motivational attitudes towards the tool, (3) importance of mobile-assisted tool to enhance grammar knowledge and finally, (4) students’ understanding of the difficulty on the instructions of the tool. To analyze the responses, the interview data were coded into 3 scales (positive, confused and negative) based on 4 questions (1 = interest, 2 = motivation, 3 = importance, 4 = difficulty).

In the interview protocol, only a few students were chosen from the experimental group who voluntarily participated in the interview session. The students were interviewed individually to investigate their experiences and opinions on the use of the tool and its design. For each participant, it took around 10 minutes and around 60 minutes in each session and in this way 5 sessions for one week to cover 30 students’ (15 males and 15 females)interview. The participants were also given time flexibility so that they can assume or anticipate the answers to the questions. They were also assured that the recorded interview session would be confidential and only be used for research purposes. The questions were semi-structured since additional questions were asked to get the detailed responses from the students.

### 3.3 Research material

The research material of this study is a mobile-assisted tense tool, which is a web-based tool for learning English present tenses. At present, despite having some internet-based learning applications for English tenses, no study is designed on the ARCS model as a motivational model [3–6]. This mobile-assisted tense tool is designed keeping in mind that it can help to motivate students in terms of bringing attention, finding relevancy and confidence and finally feeling satisfied when using it.

To develop the tool, the process started with content development. In the beginning, an outline of the content was drawn since developing effective content is essential for any kind of instructional design of learning materials. Therefore, the content on English present tense was designed based on students’ level of knowledge and to improve the achievement of a particular grammar item (English present tense). Then to check the content validity, the experts were asked to scrutinize the content before sending the storyboard to the programmer. A storyboard is an equally important part that guides every step of instructional design. After preparing the storyboard, it was given to the developer or programmer to shape it on the web-based platform. After the design was completed, it was given to the experts to check on its design. Experts of multimedia were also provided with the design and went for the pilot test.

A learning laboratory was used to conduct the research where the laboratory was well equipped with personal desktops and the tool was installed in the desktop for each student to use. The tool was also enabled in smartphones so that students could also use the tool from smart-phones. Since the tool is designed to be used in both smart-phones as well as in laptops or desktops, the tool is named a mobile-assisted tense tool. Moreover, for the easy access and measuring the performance, it was necessary to have every student his personal monitor featured with multimedia. Needless to say, it was not possible to have every student a high featured mobile phone, therefore the tool was installed in the laboratory desktops to conduct the research feasible. The tool contains four modules, which are (1) log-in module, (2) learning module, (3) exercise module and (4) self- evaluation module.

The tool contains four modules, which are 1) log-in module, 2) learning module, 3) exercise module and 4) self- evaluation module.

The tool was designed taking into consideration the motivational model for instructional design by Keller. According to the theory of motivation, any learning material must gain the attention of the students [31]. Therefore, songs and images were added as stimuli that generate interest. Few studies also applied songs and images to sustain motivation among the learners [32]. It is also postulated that music or songs are processed in the different hemisphere in the human cognitive system and therefore, concurrently provides effective feedback in language learning [33].

The tool also includes a variety of stimuli for input and notes to continue maintaining students’ interest. To establish the relevance of instructions, common examples, and real-life instances were included in the tool. The tutorial with examples is part of the learning module. The exercise module consisted of different types of questions such as fill in the blanks, multiple-choice questions, table matching, quizzes, sentence building on present tenses. To add, the benefits of completing the exercises, which was to instill students’ confidence are shown by providing the scores. It also provided the right answers if they make mistakes while filling up the answers in exercises. It explores the unknown and the tool acts as a support, which enhances the students’ capacity of thinking as part of problem-based learning system [34]. This encouraged the students to learn more and monitor their progress in performance. This part is important to build students’ confidence during the learning process. Feedback is a very important part of learning as it motivates learners to go ahead [35]. The final criterion in the model is satisfaction building. To manage students’ satisfaction, a self-evaluation part is included in the tool, where they were asked to answer questions on tenses. This part of the evaluation is an outcome of their performance. Through the scoring system of performance outcomes, students become aware of their progress that acts as a reward, which validates the importance of the tool. The questions were designed to be multiple choice and fill in the blanks.

### 3.4 Research procedures

The procedure is divided into two phases. During the first phase, students were required to sit for the 30-minute paper-based test as a pre-test to measure their knowledge on English present tense. Then, the participants were introduced to the mobile-assisted tense tool, where they were requested to use the tool for four sessions where each session was designed for one hour use. In every session students went through one part of the present tense, for instance, in the first session students learned only simple present tense and in the second session they went present continuous and likewise the other sessions continued. In the one hour duration, the students went through the instructions at the beginning of the application, then they watched the tutorials. At every criterion of the tense, there were also exercises. Exercises with answers or clue(the clue or answer is shown after the click on the options. If the right option is clicked or written then the green color appears on the option. On the other hand, if the answer was given wrong by the students the red or cross appears. Meanwhile, the right answer appears) helped them to practice and learn. The exercise part was designed with different kinds of practice such as fill in the blanks, multiple-choice questions, short quizzes. After that, they had to go for the self-evaluation process through which they could see the score of their performance answering the questions without a clue. This is how the students spent the treatment phase. They went through the practice after their two-hour scheduled classes ended. The tool was installed in computers in the English language laboratory. The second phase of the study is the evaluation part, which consisted of students’ feedback on the use of the tool. The IMMS survey was administered to the students after they used the tool for one hour, which took 15 minutes to be collected. Afterward, they were then asked to sit for another paper-based test, which was a post-test. The purpose of the post-test was to investigate the differences in the outcome after using the tool. The post-test was also 30 minutes. After the administration of both, the IMMS and paper-based test, a semi-structured interview was conducted with 30 students, which took around 2 hours. Since this study followed a sequential mixed-method research approach, this interview session was conducted after the quantitative data collection i.e. IMMS and paper-based tests. The duration of collecting feedback from the interview was about 30 minutes and it took 2 weeks for data collection.

## 4 Research findings

The findings were presented based on data collected from the (1) Instructional Material Motivation Survey (IMMS), (2) the paper-based test, and (3) the interview protocol.

### 4.1 Instructional Material Motivation Survey (IMMS)

To examine student motivation, students were asked to use the tool and their motivation in terms attention(ATT), relevance(REL), confidence(CON) and satisfaction(SAT) was measured using the IMMS.

Descriptive statistics for each item of the four criteria of the IMMS were summarized in Table 2. For all four criteria, the statistical outcome indicated a strong support of the instruction design of the tense tool. Students’ responses in IMMS were illustrated in mean and standard deviation.

**Table 2:** Means and standard deviation of the IMMS items.

*Criteria*	*Item*	*InstructionalMaterialMotivationalSurvey*	*Mean*	*SD*
Attention	1	There was something interesting at the beginning of the tool that got my attention.	4.25	0.63
	8	The variety of exercises, illustrations, in the tool, helped keep my attention	4.08	0.84
Relevance	3	There was stories, pictures or examples in the tool that showed me how this material could be important to some people	4.06	0.73
	6	The content of this learning material is relevant to my interests	4.00	0.75
	9	I could relate the content of this tense learning tool to things I have seen, done, or thought about my own life	4.15	0.71
Confidence	4	Completing the exercises successfully was important to me	4.09	0.71
	5	As I worked on this lesson, I was confident that I could learn the content	4.23	0.78
Satisfaction	2	Completing the exercises in this lesson gave me a satisfying feeling of accomplishment	3.97	0.86
	7	I really enjoyed studying this tense learning tool	3.97	0.85
	10	It was a pleasure to work on such a well-designed learning tool	4.11	0.87

Attention is the first aspect that learners must gain in order to maintain their motivation for the learning tool (Keller, 1983). In this study, IMMS includes two items (ATT1and ATT8) which inquired about the interesting aspects and variation of the tool design and its instructions. ATT1 inquired about the perceptual arousal aspect to gain learners’ attention (M = 4.17), and ATT8 asked about various interesting aspects applied in the design of the instruction (M = 4.08). This result showed that the tense tool was able to catch their attention, which indicated the strength of the tool in creating high motivation among learners.

The second criterion assessed was the relevancy of the content, activities and exercises contained in the tool. It has to be real or close to them so that they feel the real execution of learning. REL3 asks about illustrations, examples or exercises used to design the instructions (M = 4.06). REL6 (M = 4.00) and REL9 (M = 4.15) put insight on how much the learning instruction was meaningful and relevant to the users.

Confidence requires learners to maintain their motivation on the learning material. In the IMMS, CON4 (M = 4.09) and CON 5 (M = 4.23) viewed learners’ confidence level as a way to motivate them in using the tool. The last criterion of the ARCS motivational model was the satisfaction in completing the learning process. When students feelt satisfied with the learning instructions, the instructional design was then successful. To assess satisfaction, the IMMS included 3 items as SAT2, SAT7 and SAT10. The means for the items were respectively 3.97, 3.97 and 4.11.

The IMMS data was also analyzed according to gender, which illustrated in Table 3. The data illustrated a difference between male and female students in terms of their motivational aspect where the male students were more motivated in using the tool as compared to the female students.

**Table 3:** Means and standard deviation of the IMMS items according to gender.

Gender		*Attention*	*Relevance*	*Confidence*	*Satisfaction*	*Total* (*Motivation*)	(*p*)
Male (n = 39)	Mean	4.19	4.10	4.22	4.05	4.14	0.01
	*SD*	0.70	0.72	0.72	0.84	0.74	
Female (n = 76)	Mean	4.13	4.02	4.11	4.00	4.06	
	*SD*	0.86	0.75	0.85	0.78	0.81	
Overall (n = 115)	Mean	4.16	4.06	4.16	4.02	4.10	
	*SD*	0.78	0.73	0.78	0.86	0.78	

The result showed that the attention level among the male students was greater than that of the female students (M = 4.19 > 4.13). Next, the male students indicated that the learning tool was more relevant (M = 4.10) compared to female students (M = 4.02). Student confidence and satisfaction of the learning tool were also higher for males than females (M = 4.22 > 4.11) and (M = 4.05 > 4.00). Here, the result suggests that the total motivation was higher for male students (M = 4.14) than female students (M = 4.06). The findings indicated that males were highly motivated in all four criteria on the usage of the tool. To ensure the difference in whether the outcome of male and female total motivation was significantly varied or not, a T-test was run.

From Table 3, it was observed that there was a significant difference of motivational criteria between male (M = 4.14) and female students (M = 4.06), t = 5.66, p < 0.01. From the T-test, it was observed that males were higher in all criteria of motivation e.g. attention, relevance, confidence and satisfaction.

### 4.2 Paper-based result

Students’ learning improvement was derived from the findings of the paper-based test. It was necessary to analyze the learning improvement on paper-based outcome from pretest to post-test. The finding of the pretest to post-test of experimental group is shown in Table 4.

**Table 4:** Means and standard deviations comparing the pre-test and post-test of the paper-based result.

*Condition*	*Mean*	*Std*.*Deviation*	*Std*.*Error*
Pre	8.52	1.74	0.17
Post	14.17	1.90	0.18

A paired samples *t*-test was run to investigate the difference between the outcome of paper-based test on pre intervention and post intervention period. Table 5 illustrated that outcome of the paper-based test indicated a significant difference from pretest (M = 8.52, SD = 1.74) to post-test (M = 14.17, SD = 1.90), t (103) = -23.53, *p* <0.05(two-tailed). The mean increased in post-test scores was 2.27 with a 95% confidence interval ranging from -6.12 to -5.17. The eta squared statistic (.70) indicated a large effect size.

**Table 5:** Means and standard deviations of gender based paper-based results.

*Gender*	*Mean*	*Std*.*Deviation*	*p*
Male	14.86	1.52	0.05
Female	9.20	1.85	

An independent *t*-test was run in order to investigate the gender based outcome of the students in the performance of paper-based result. Result showed that the males had higher means of score in post-test (M = 14.86, SD = 1.52) than females (M = 13.44, SD = 2.11) that they had in pre-test respectively males (M = 9.20, SD = 1.85) and females (M = 7.90, SD = 1.37). There were significant differences between the males and females in their learning performance both in pre and post- test as *p* < 0.05.

### 4.3 Interview result

#### 4.3.1 Students’ opinions on the tool utilization

**Fig 1:**
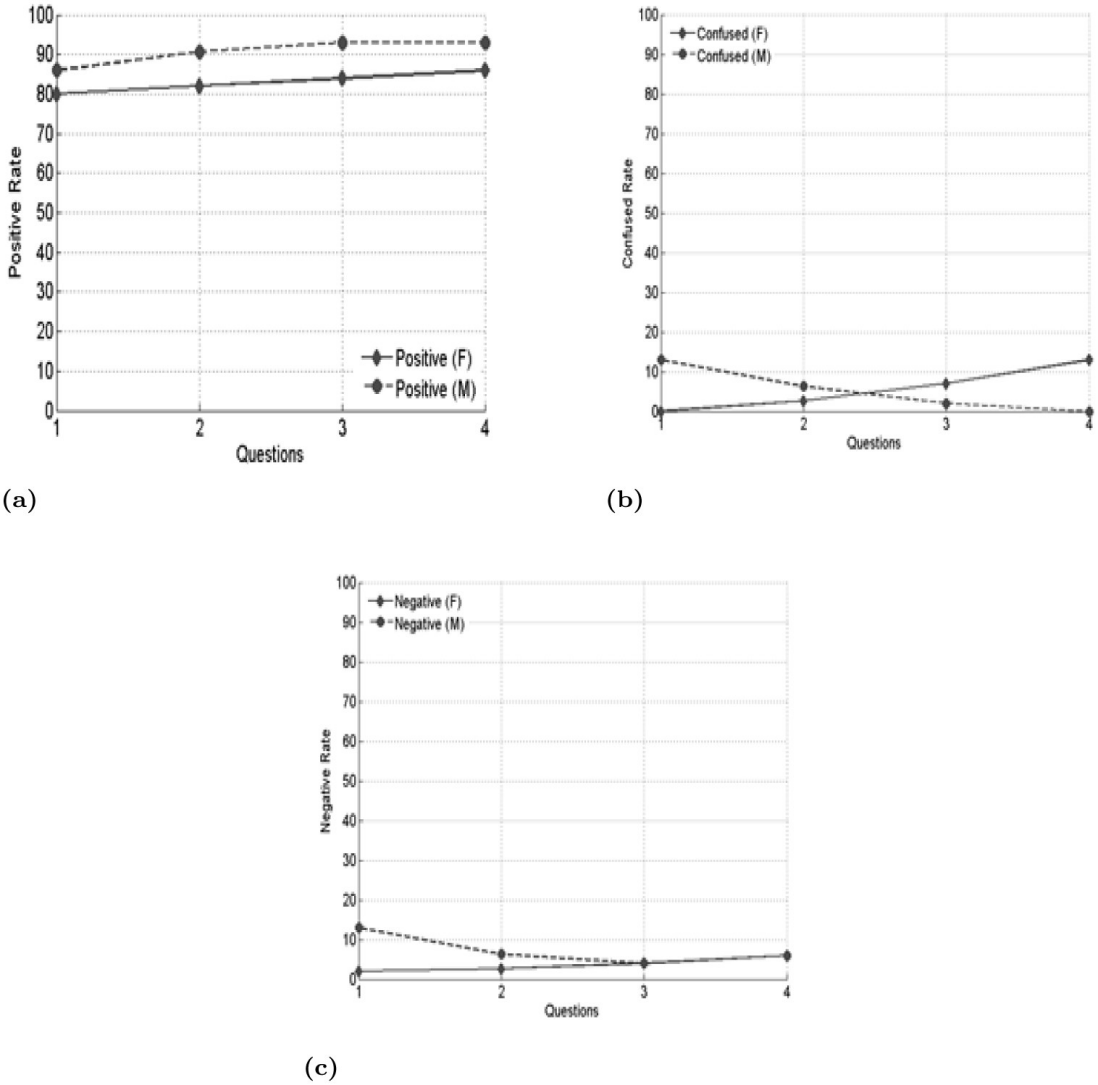
Interview outcome of the students.

The purpose of the interview was to gather students’ opinions regarding their interest in using the tool, their motivational attitude, the importance and the difficulty level of the tool. The results focused on the positive, confused and negative attitude rate of the students towards the tool. The total ratio of male and female interview outcomes was illustrated in Fig 1, which shows that males were more positive in terms of their interest and motivation to learn. Females indicated slightly lower in their interest and motivation as compared to males.

The total ratio of male and female interview outcomes was illustrated in Fig 1, which illustrated that males were more positive in terms of their interest and motivation to learn. Females were a bit lower in this aspects compared to males.

Fig 1 was separated into three line charts differentiating three different attitudes towards the use of the tool. Part (a) depicted students’ positive attitude towards the tool where X axis showed the items of the interview questions and Y axis was the representation of their level of positive attitude. Similarly, line chart (b) and (c) are presented in this figure respectively to suggest students’ confused and negative opinion on the interview questions.

This result showed that among the 15 male students, 86% of the students were interested to use the tool (Q1), 93% of the students expressed positively regarding the motivational attitude towards the tense learning tool (Q2), and the importance the tool for learning (Q3), but most of them also agreed that there are no design difficulties in instructions of the tool (Q4). On the other hand, among 15 females, 80% of the females replied positive for interest on the tool (Q1) and 86% of the student addressed positive for motivational perspective of the tool (Q2). Regarding the importance of using the tool (Q3), 80% of the female students agreed for such necessity to have such a learning tool and 86% replied positive on “no design difficulty” on the tool.

To illustrate the outcome of confused attitude, it was noticed that a few numbers of students were confused with Q1 and the percentage of confusion for male was 13%. Conversely, females were not confused at all on this item. Even for other items namely Q2, Q3 and Q4, both the males and females were found less confused with motivation, importance and designing difficulty respectively.

The result of the negative attitude on the tool was also important in order to investigate and evaluate the design of instructions of the tool. Since a few percent (male = 13% and female = 2%) replied negatively to the item (Q1), it was revealed that students found the design of the instruction as interesting. They specifically commented that the introduction to the illustrations, for example, exercises in multimedia attracted their attention. In the motivation statement (Q2), only 6% males and females gave negative statements as the tool. According to them, the tool was not motivating and using technology is not important in the class. Items Q3 and Q4 revealed that no male or female replied negatively to them. Both genders were equally contended that the tool was important and designing of the instructions of the tool is not difficult to understand.

The outcome of the study is equally important in the aspect of motivational outcome and attitudes of the learners towards the tense tool. The results indicated positive outcomes of the design of the initial version of the tool. Attention, relevance, confidence and satisfaction are high in the aspect of instructional design of the tool. Males are more positive in their attitudes towards the design of the tool while females are lesser positive in their opinion of the tool design. This guided for the following process of how the tense tool can be further developed.

#### 4.3.2 Students’ opinions on impacts of using the tool on motivation

The impacts of using the tool on student motivation is also examined in the student interviews. The analysis yields two themes; (1) motivation outcome, and (2) learning outcome. To explore students’ motivation on the instructional design and usability of the tool, they are asked to share their experience. The students viewed their motivation of using tool positively and satisfactorily. Most of them felt motivated about the design of the tool i.e., illustration, exercises, examples and gaming which stimulated and sustained their attention as well as feeling satisfied. They felt the tool enabled them to learn better.

- **PR01**: The tool is motivational because it gives me lot of examples that match with my regular task.
- **PR02**: The tool helps me to motivate and feel confident in learning present tense.
- **PR05**: I feel very much motivated because earlier I did not know about the differences between present and present tense but now I am confident about the use.
- **PR11**: it is motivating because I think I want to improve in grammar importantly in present tense which I am able to do with the use of this tool.
- **PR14**: I feel motivated because now I know where my fault is and what to correct. In few cases, exceptions were found on the motivation outcome of students who claimed the tool was not motivational.
- **PR09**: It is very complicated because it is my first time to use learning application.
- **PR13**: I am little bit motivated because I think it is helpful for the kinds who does not know the use of English tense. Attention and satisfaction of the students are two important factors of motivation as discussed in the ARCS model. To query about the opinion on attention and satisfaction about the design of the tool students were asked about how much they felt the tool attractive and satisfied with the design of the tool.
- **PR01**: I feel the design interesting, not boring.
- **PR03**: It catches my attention and I feel satisfied
- **PR04**: It takes my attention and I feel satisfy as enjoying the filling up exercises and more importantly the game part is interesting.
- **PR11**: I feel satisfied because it shows the scores after one finishes the evaluation part and gives the feedback by showing level of uses.

Relevancy is an important criterion of ARCS model. Students’ replies were optimistic on the design of the content. Most of the students felt the content of the tool was relevant to real life.

- **PR02**: I think the content has relevant to the real life
- **PR05**: The content is relevant and we can relate it to daily life communications Regarding the confidence on the tool, most of the students felt confident and agreed that they can use present tense more correct than the time before they could.
- **PR04**: I feel confident that this tool is useful for communication.
- **PR07**: The tool is effective as it has built confidence within me about the proper use of English tense. Responses to this variable represented the interviewees’ viewpoints and perceptions about the relevancy of the examples and illustrations of the design to real life. All students agreed the illustrations and examples do matches with their real life exchanges of conversations and have the relevancy.

#### 4.3.3 Students’ opinions on tools utilizations on learning performance

The interviews also investigated students’ opinions on the effects of using the tool on their learning achievement of the present tenses. This focused on the outcome on how much using the tool could contribute for better performance and knowledge construction in present tenses. Few students replied that the tool helped them to know more about present tense and use them properly in the present context they require than before they could use.

- **PR07**: I am confident that this tool can help to enhance our knowledge on present tense.
- **PR12**: Yes, it can enhance the knowledge of the students on present tense.
- **PR14**: It is helpful to learn present tense. Besides lectures, integrating technology is helpful. As such, adapting technology into classrooms has become popular nowadays. Upon investigating the opinions about the usefulness of adopting technology beside classroom lectures, it was found that students were very much optimistic
- **PR04**: I think it is very important to integrate the tool in the classroom.
- **PR06**: This tool can be used in the classroom to teach English tense more intensively.
- **PR08**: I think this tool is helpful in this new era for learning with the help of technology.
- **PR09**: I am very positive that this tool can be used in the classroom to learn present tense beside the class lecture.

This finding from a semi-structured interview relates to the theoretical assumption of the ARCS model that students become highly motivated if the instructional design of the material follows four components of motivation. Instructional design based on the motivational model is always a supporter of effective learning as supported by some studies [36, 37]. Using the strategies suggested in the ARCS model i.e., perceptual arousal for attention, bringing variability in the tutorials as well as games helped the students growing interested in the tool. Students with their intrinsic motivation have the instinct to have competence and self-regulation action. This action comes from experience which includes exploration, creativity curiosity, and asking the question. Likewise in this tool, the student could handle the action of their learning activities the following skills: reading and understanding the illustrations and explanations of different definition, usage, and exceptions, the procedure to accomplish the exercises (i.e., helps to practice with different examples), evaluating their self-progress through feedback messages [38]. On the other hand, a few studies showed that students with low motivation have a low academic achievement [39].

The students also felt the design was well organized and interesting. They believed that technology-based grammar learning was a challenging system with exploiting multimedia. Therefore, it needs more attention from the researchers to design a more effective way so that it can enhance knowledge. Grammar learning experience not only increases the possibility to do perform in academic perspective but also enables them to implement this knowledge in communications as well. Some of the students even expressed that they had before a little knowledge in English Present tense but with the usage of the tool, they learned a lot about the Present tense. Few interviewees replied that such a motivational design can increase their interest in grammar which is very much effective in the development of every language skill (speaking, listening, writing and reading). Additionally, because of technology-based learning materials, the cost of formal education may be decreased greatly. It can also be utilized in the home environment which is largely beneficial for the students who often miss the classes on universities. They can get access at home to know and analyze the tutorial, after finishing there is a feasibility of using the exercises.

## 5 Discussion

The findings of IMMS illustrated that the students positively believed the use of the tense learning tool motivated them to learn English tenses. The outcome of the T-test was relevant to the interview result which ultimately showed that the learners founded the instruction design equally attractive. For examples, illustrations were relevant to the real context; all the feedback process, such as rewards to the completion of the levels of modules, built up confidence and finally satisfied them in a standard level. The data establishes the suitability of applying the ARCS model for the development of a mobile-assisted tense learning tool. In comparison to other studies [40, 41] which applied the ARCS model for designing learning instructions for motivating different level of learners, it is evident that the application of the ARCS model can improve students’ motivation level in learning experiences

According to the ARCS model, the first criteria of motivating learners is bringing attention, which must be maintained through perceptual, inquiry arousal or variability in the instructions. In this tense learning tool, we applied different approaches to attract students. We used songs and animation as perceptual arousals to introduce tense. We also maintained variability to continue and sustain learners’ attention by posing a story on tense. The opinions from the interview confirmed that the tool was interesting because it used music and videos to bring attention to tense learning. Some students also pointed out that it was an innovative idea to put the story to know sentence structures of tense. It was evident from the IMMS result also as ATT (M = 4.16). This result can be supported by other results investigated in separate studies [40]. A previous study was conducted to investigate the motivation for language learning of polytechnic students who were given a multimedia e-book (mE-book). The content of the multimedia E-book was filled with graphics, colorful texts, animations, and sound. It is proven from the research that the learning materials designed with animation and graphics can convey more information and also attract the learners [41]. Another research also revealed that using video to motivate learners for the learning system proved effective. The video system helped to specify the visual and pictorial learning materials that reinforce learning and this made students become more engaged and acquired more information [42].

The second criterion was relevance, which is important for designers to include in the instructions because it is an effective learning system. Designers should be able to match the illustrations to be relevant to real life. Generally, learners will be more motivated to learn if they perceive that the new knowledge or skill will help them achieve a goal in the present or future [43]. The result of the IMMS in this present study demonstrated that the students had REL (M = 4.06) since students tended to be most interested in the content of the instructions, which had some connections to their prior experiences and interests. The result can be compared with other studies that were done on English as a foreign language (EFL) students where they agreed that the design of teaching content was important and relevant to their needs for learning the English language [44].

Users of any learning materials should have confidence in using a particular learning tool. Confidence highlights the importance of students’ feeling confident in their ability to succeed. Keller argues that letting the learners know what is expected of them, is one of the simplest ways to help instill confidence. In this present research, the confidence of the learners has been strong that focused on the positive outcome of the motivational instruction design, which was evident from the outcome of IMMS (CON M = 4.16). The students had the feeling that completing the tasks in the instructions can be useful to them and they can learn tense through this tool. These statements were further strengthened through opinions given in the interview. They felt this tool was important to bring confidence to them. The exercises also helped them to judge their improvement of knowledge level. Some other studies were done to know the motivation of the students where confidence was one of the main criteria [29, 30] found using the ARCS model can help to enhance confidence.

Satisfaction emphasizes the contribution of feeling satisfied after a learning experience for motivation to continue. The outcome of this study confirmed the level of satisfaction of the students (SAT M = 4.02), which suggests that students got proper feedback and evaluation by using the tool after the completion of each tense learning. Here, the evaluation part was designed so that the learners can feel that they were awarded if they were successful, or gotten lagged if they were unsuccessful in getting a good score. This reward method is an extrinsic approach that also enhances the learners’ desire to do better in the learning outcome.

It was noticed from the above discussion that the tense learning tool had a positive impact on the learners’ motivation. It was revealed that using the ARCS model in designing a tense learning tool gained attention, brought relevancy in learning materials which strengthened students’ confidence and ultimately, satisfying them. The interview results also showed that students approved this tool as an important, interesting and motivating way to learn English tense. It was also the purpose of the study to measure the progress achieved in learning performance, specifically on English present tense after the use of the tool. The paper-based test was aimed to measure the improvement of grammar learning achievement of the students. The outcome from this instrument provided a positive result where students performed better in the post-test than the pretest. This improvement in the paper-based result proved that students increased knowledge on the present tense that they had before.

It was evident from different studies where students who were motivated in their learning materials, go through better learning performance. It was further analyzed that students gained more knowledge than the users of paper-pen based grammar classes. In this study, it was evident that students gained knowledge in the present tense after using the tool. They felt the tool helped them to know the rules more easily for extracting the examples from daily activities. The paper-based post-test (M = 14.17, SD = 1.90) indicated a positive outcome of the students, which was further supported by the interview. The interviewed students shared that they had previously little knowledge on present tense as compared to after using the tool where they knew then how to use present tenses in writing as well as speaking.

This study is about an instructional design that is based on the ARCS model to induce motivation to the learners, intrinsically and extrinsically. Since the new experience of grammar learning using a mobile device with encouraging and interesting instructional design, these students felt more enthusiastic to work on it. They declared such motivation to be not merely intended to pass the examination but also to practice the tool to improve their knowledge in the present tense. The fact is also evident from the quotes of Keller, who reported that individuals with intrinsic motivation, engage in tasks for the pleasure that the students seek challenges where active participation comes naturally [45]. He further mentioned that proper instructional design can be effective to build intrinsic motivation in the students who can have an external goal, to seek challenges in the learning.

Now, considering the four constructs of the ARCS model, the illustrations with multimedia stimulate students’ curiosity and intrinsic motivation. Because of their curiosity and experience using these techniques of answering different quizzes, the ability to solve problems, getting reward quotes at the end of each level, the students focus on increasing relevance and confidence where ultimately, the whole process satisfies them towards meaningful learning. These strategies allow students to have some control over the content, increasing their intrinsic motivation. The outcome of the semi-structured interview thus revealed their increased motivation and the ability to improve their pre-existing knowledge on the present tense after using the tool.

Also, from the study, it was revealed that the outcome of using the tool was more positive for males than females. There was no obvious reason to justify the outcome of the paper-based test or motivational outcome was better for male students. However, limited research was found to focus on performance analysis based on gender differences where most of the cases females outperformed the males in language learning. Anxiety could be one of the reasons that worked for the females for which they could not perform higher as males. This kind of outcome in language learning was also found in the studies where females were more anxious than males at the tertiary level of students [46, 47].

To motivate learners, Keller’s ARCS model was utilized to design and develop the tense tool and its instructions. To stimulate and sustain learner motivation, different types of audiovisual instructions were included in this tool. Motivational learning should consider the learners’ attention, relevancy to the instructions, confidence and satisfying their needs. The findings of the study confirmed that the tense tool can motivate the learners on a great scale. It is observed that the implementation of the ARCS model in technology-based language learning was a successful experiment in the present research era. The outcome confirmed students’ level of concentration in their learning and ultimately, made effectively as well as gave positive differences to their language learning achievement. It was more challenging in educational research to get the same positive feedback in mobile-assisted language learning. Through mobile and grammar learning gained few decades to investigate the outcome on learners, it was an unexplored area to know the impact of any mobile-assisted tense tool on learners’ motivational outcome and improved learning performance on English present tense. The present research work thus attempted to achieve this aim and has been successful to get an effective result.

## 6 Limitation of the study and recommendations

The main limitation of the study was the sample size. The study was a preliminary investigation, thus limited students were considered for the sample. The sampling was done randomly but simple random sampling was time-consuming and rather costly. In this study, one of the limitations of using random sampling was time-consuming, as selecting the students from a large population and collecting data for their background knowledge was difficult. Moreover, the outcome of other variables was not considered i.e. the need to know learning achievements after using the tense learning tool. There could be in-depth analysis to measure the correlation between motivation and learning achievement, or inferential statistical reporting of the paper-based test from pre-condition and post-condition of learning achievement of the students. Therefore, the generalization of the results was not fully considered. A generalization is an act of inferring the broad outcome from a particular observation. In this study, the cautious approach was maintained because the sample size was not big according to the population. Moreover, due to random sampling, the background knowledge of the students was not analyzed in deep. In addition, for the qualitative part, the generalization of the perception was not attempted. In addition, students were given the tool to use only for one hour in four sessions, which was a limited time to explore further on the tool. Frequent practice of the exercises and evaluations could be more effective. Therefore, time was a factor, which needed to be considered. In addition, the practice of the tool was done using computer desktops in the language laboratory due to a lack of personal smartphones. Practice with personal mobile devices could have been more flexible in terms of practicing the exercises or going through the tutorial with the learners.

For future research, the ARCS model can be a useful model in bringing motivation through instruction design in different aspects of grammar. This model may be useful to assess classroom material design as well, such as the multimedia instructions which can bring attention, relevance, confidence, and satisfaction. Moreover, the tool can be used in other samples such as in other classes which require the learning of English tenses. In addition, future work should further investigate the impact of online learning incorporating the proposed mobile tools on learning outcomes, using a range of methods, participants, and courses.

## Supporting information

S1 File (ZIP)
