# Supplementary figures and images for: Measuring student motivation on the use of a mobile assisted grammar learning tool
Source: PLoS One. 2020 Aug 28;15(8):e0236862. doi: 10.1371/journal.pone.0236862 (PMC7454966; doi:10.1371/journal.pone.0236862)

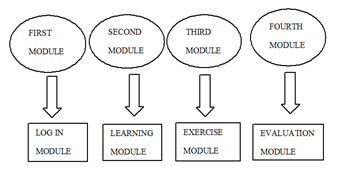

Supplement: S1 File — (ZIP) [file pone.0236862.s001.zip › Latex_Source_File/fig1.png]

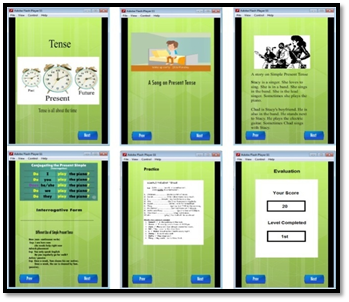

Supplement: S1 File — (ZIP) [file pone.0236862.s001.zip › Latex_Source_File/fig2.png]

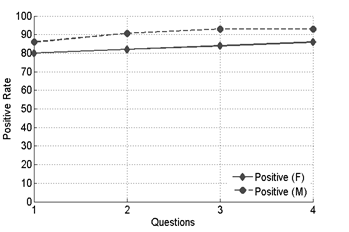

Supplement: S1 File — (ZIP) [file pone.0236862.s001.zip › Latex_Source_File/fig3_a.png]

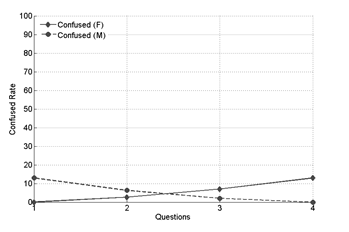

Supplement: S1 File — (ZIP) [file pone.0236862.s001.zip › Latex_Source_File/fig3_b.png]

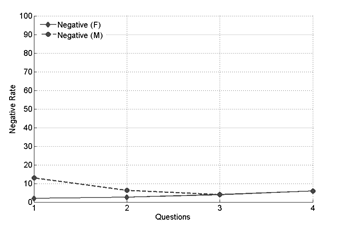

Supplement: S1 File — (ZIP) [file pone.0236862.s001.zip › Latex_Source_File/fig3_c.png]

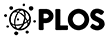

Supplement: S1 File — (ZIP) [file pone.0236862.s001.zip › Latex_Source_File/PLOSlogo.png]

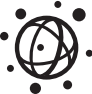

**PLOS**

**SUBMISSION**

Supplement: S1 File — (ZIP) [file pone.0236862.s001.zip › Latex_Source_File/PLOS-Submission-eps-converted-to.pdf]
